# Supplementary material for: Distribution and Chemistry of Phoenixin-14, a Newly Discovered Sensory Transmission Molecule in Porcine Afferent Neurons
Source: Int J Mol Sci. 2023 Nov 23;24(23):16647. doi: 10.3390/ijms242316647 (PMC10706208; doi:10.3390/ijms242316647)
Supplement: Supplementary file 1 [file ijms-24-16647-s001.zip › Figure S1.pdf]

| #1 | b⁺ | b²⁺ | Seq. | y⁺ | y²⁺ | #2 |
|---|---|---|---|---|---|---|
| 1 | 116.03422 | 58.52075 | D | | | 14 |
| 2 | 215.10263 | 108.05496 | V | 1468.79471 | 734.90099 | 13 |
| 3 | 343.16121 | 172.08424 | Q | 1369.72630 | 685.36679 | 12 |
| 4 | 440.21397 | 220.61063 | P | 1241.66772 | 621.33750 | 11 |
| 5 | 537.26674 | 269.13701 | P | 1144.61496 | 572.81112 | 10 |
| 6 | 594.28820 | 297.64774 | G | 1047.56219 | 524.28473 | 9 |
| 7 | 707.37227 | 354.18977 | L | 990.54073 | 495.77400 | 8 |
| 8 | 835.46723 | 418.23725 | K | 877.45666 | 439.23197 | 7 |
| 9 | 934.53564 | 467.77146 | V | 749.36170 | 375.18449 | 6 |
| 10 | 1120.61496 | 560.81112 | W | 650.29329 | 325.65028 | 5 |
| 11 | 1207.64698 | 604.32713 | S | 464.21397 | 232.61063 | 4 |
| 12 | 1322.67393 | 661.84060 | D | 377.18195 | 189.09461 | 3 |
| 13 | 1419.72669 | 710.36698 | P | 262.15500 | 131.58114 | 2 |
| 14 | | | F-Amidated | 165.10224 | 83.05476 | 1 |

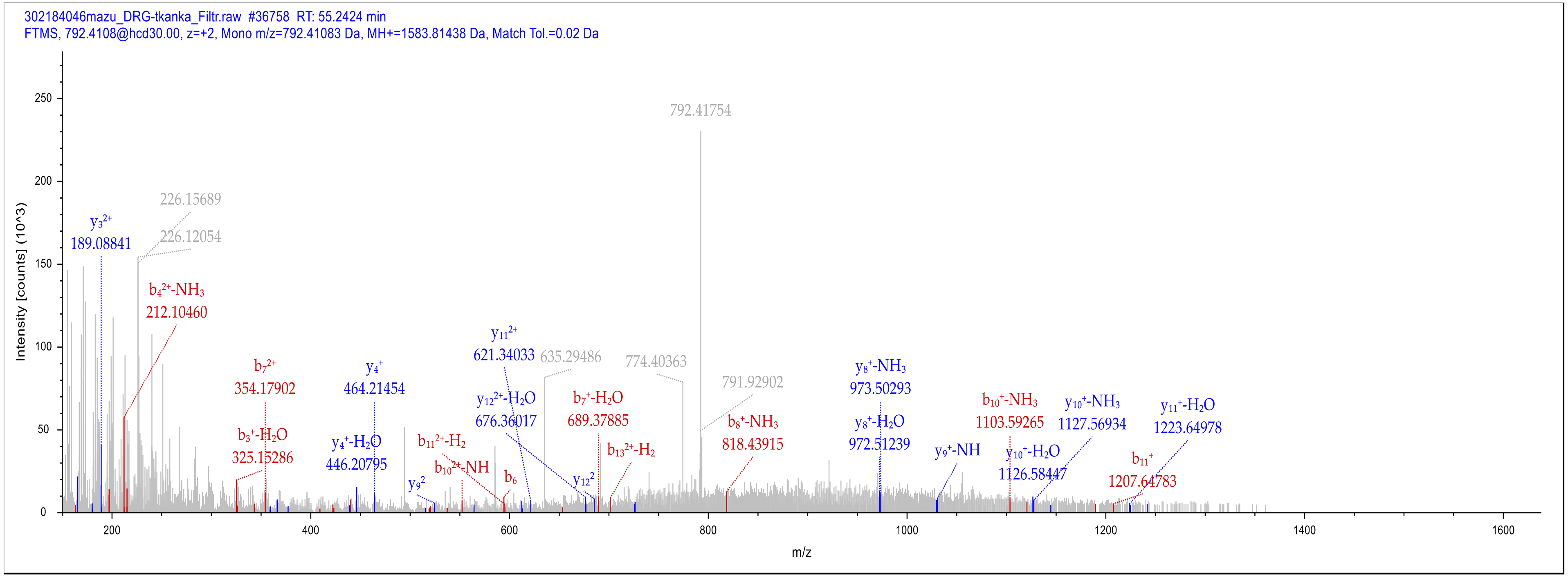

**Figure S1. The list of mass spectra of each amino acid and fragmentation spectrum – amidated (-0.98402 Da) that makes up PNX, detected in L5 DRG from animal nr 1 (DRG1)**

Phoenixin fragmentation spectrum – amidated (F)

Sequence: DVQPPGLKVWSDPF, F14-Amidated (-0.98402 Da)
Charge: +2, Monoisotopic m/z: 792.41083 Da (-3.64 mmu/-4.59 ppm), MH+: 1583.81438 Da, RT: 55.2424 min,
Identified with: Sequest HT (v1.17); XCorr:2.50,
Fragment match tolerance used for search: 0.02 Da
Fragments used for search: -$H_2O$; y; -$NH_3$; y; b; b; -$H_2O$; b; -$NH_3$; y
