# Supplementary material for: Distribution and Chemistry of Phoenixin-14, a Newly Discovered Sensory Transmission Molecule in Porcine Afferent Neurons
Source: Int J Mol Sci. 2023 Nov 23;24(23):16647. doi: 10.3390/ijms242316647 (PMC10706208; doi:10.3390/ijms242316647)
Supplement: Supplementary file 1 [file ijms-24-16647-s001.zip › Figure S3.pdf]

#1	b^+	b^{2+}	Seq.	y^+	y^{2+}	#2
1	116.03422	58.52075	D			14
2	215.10263	108.05496	V	1468.79471	734.90099	13
3	343.16121	172.08424	Q	1369.72630	685.36679	12
4	440.21397	220.61063	P	1241.66772	621.33750	11
5	537.26674	269.13701	P	1144.61496	572.81112	10
6	594.28820	297.64774	G	1047.56219	524.28473	9
7	707.37227	354.18977	L	990.54073	495.77400	8
8	835.46723	418.23725	K	877.45666	439.23197	7
9	934.53564	467.77146	V	749.36170	375.18449	6
10	1120.61496	560.81112	W	650.29329	325.65028	5
11	1207.64698	604.32713	S	464.21397	232.61063	4
12	1322.67393	661.84060	D	377.18195	189.09461	3
13	1419.72669	710.36698	P	262.15500	131.58114	2
14			F-Amidated	165.10224	83.05476	1

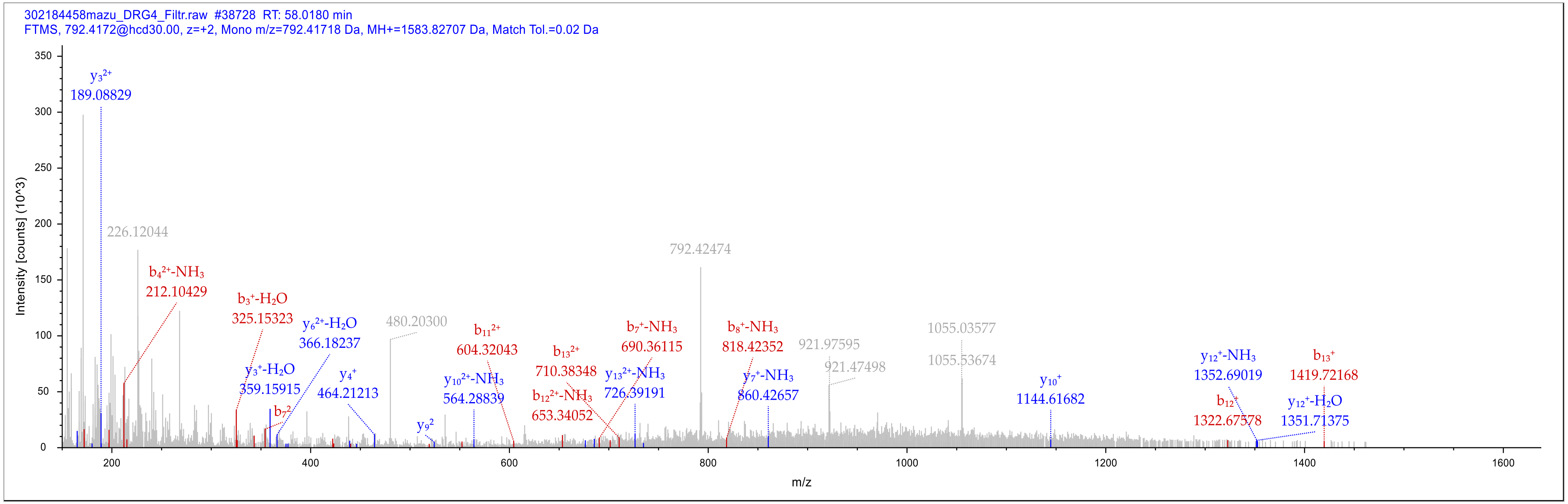

Figure S3. The list of mass spectra of each amino acid and fragmentation spectrum – amidated (-0.98402 Da) that makes up PNX, detected in L5 DRG from animal nr 3 (DRG3).

Phoenixin fragmentation spectrum – amidated (F)
Sequence: DVQPPGLKVWSDPF, F14-Amidated (-0.98402 Da)
Charge: +2, Monoisotopic m/z: 792.41718 Da (+2.71 mmu/+3.42 ppm), MH+: 1583.82707 Da, RT: 58.0180 min,
Identified with: Sequest HT (v1.17); XCorr:2.24,
Fragment match tolerance used for search: 0.02 Da
Fragments used for search: -H_2O; y; -NH_3; y; b; b; -H_2O; b; -NH_3; y
